# Transcriptomic analysis of *Porphyromonas gingivalis*‐infected head and neck cancer cells: Identification of PLAU as a candidate prognostic biomarker

**DOI:** 10.1111/jcmm.18167

**Published:** 2024-02-16

**Authors:** Masakazu Hamada, Hiroaki Inaba, Kyoko Nishiyama, Sho Yoshida, Yoshiaki Yura, Michiyo Matsumoto‐Nakano, Narikazu Uzawa

**Affiliations:** ^1^ Department of Oral & Maxillofacial Oncology and Surgery Osaka University Graduate School of Dentistry; ^2^ Department of Pediatric Dentistry Okayama University Graduate School of Medicine, Dentistry and Pharmaceutical Sciences Okayama Japan

**Keywords:** head and neck squamous cell carcinoma, migration, *P. gingivalis*, PLAU, RNA sequencing, TCGA

## Abstract

Periodontal disease is a risk factor for head and neck squamous cell carcinoma (HNSCC), and *Porphyromonas gingivalis*, a major periodontal pathogen, has been identified as a specific and potentially independent microbial factor that increases the risk of cancer mortality. Gene expression in HNSCC due to *P. gingivalis* infection and how changes in gene expression affect the prognosis of HNSCC patients are not clarified. When *P. gingivalis* was cultured with HNSCC cells, it efficiently adhered to these cells and enhanced their invasive ability. A transcriptome analysis of *P. gingivalis* ‐infected HNSCC cells showed that genes related to migration, including CCL20, CITED2, CTGF, C8orf44‐SGK3, DUSP10, EGR3, FUZ, HBEGF, IL1B, IL24, JUN, PLAU, PTGS2, P2RY1, SEMA7A, SGK1 and SIX2, were highly up‐ or down‐regulated. The expression of up‐regulated genes was examined using the expression data of HNSCC patients obtained from The Cancer Genome Atlas (TCGA) database, and the expression of 5 genes, including PLAU, was found to be higher in cancer tissue than in solid normal tissue. An analysis of protein–protein interactions revealed that these 5 genes formed a dense network. A Cox regression analysis showed that high PLAU expression levels were associated with a poor prognosis in patients with TCGA‐HNSCC. Furthermore, the prognostic impact correlated with tumour size and the presence or absence of lymph node metastasis. Collectively, these results suggest the potential of PLAU as a molecular prognostic marker in HNSCC patients. Further in vivo and in vitro studies are needed to verify the findings of this study.

## 1 INTRODUCTION

Head and neck cancer refers to cancers that develop in the hypopharynx, oropharynx, lip, oral cavity, nasopharynx or larynx, and is the sixth most common malignancy worldwide.^1^, ^2^ Head and neck squamous cell carcinoma (HNSCC) accounts for 90%–95% of head and neck cancers.^3^ The main risk factors for HNSCCs are long‐term smoking (including cigarettes, cigars and pipes), alcohol consumption and infection with high‐risk types of human papillomavirus (HPV).^4^, ^5^

In recent years, microorganisms have also been implicated in carcinogenic mechanisms. *Helicobacter pylori* infection is the strongest known risk factor for gastric cancer and precedes cancer development.^6^, ^7^, ^8^ Periodontitis has been strongly associated with orodigestive cancer mortality, while *Porphyromonas gingivalis*, a major periodontal pathogen, was identified as a specific and potentially independent microbial factor that increases the risk of orodigestive cancer mortality.^9^, ^10^, ^11^ In addition, *Fusobacterium nucleatum*, which plays a role in periodontal disease, has been shown to play a role in the development and progression of colorectal cancer.^12^, ^13^ We previously reported that *P. gingivalis* induced the production of the precursor form of matrix metalloproteinase‐9 (proMMP‐9) via proteinase‐activated receptor (PAR)‐related pathways, after which proMMP‐9 was activated by gingipains to enhance the cellular invasion of HNSCC cells,^14^, ^15^, ^16^ while *F. nucleatum* was not.^14^, ^16^ Furthermore, Ha et al. indicated the involvement of *P. gingivalis* infection in promoting the invasive potential of oral squamous cell carcinoma (OSCC) cells via the up‐regulation of IL‐8 and MMPs.^17^ However, it currently remains unclear whether the only relevant genes are those involved in signalling pathways, such as MMPs, ERK, p38 and PAR2/NFkB. It is not clear how *P. gingivalis*, with its ability to promote the invasive potential of HNSCC cells, affects the expression of other cellular genes involved in cell motility, that is, migration and invasion.

Recent studies revealed a major advance in consolidating the findings from extensive genetic analyses of HNSCC.^18^, ^19^, ^20^, ^21^, ^22^ In 2015, the Cancer Genome Atlas (TCGA) Network reported the comprehensive genomic characterization of HNSCC.^22^ The contribution of a broader landscape and frequency of chromosomal alterations and mutations as well as gene expression profiles to the aetiology, prognosis and treatment resistance of HNSCC was demonstrated by TCGA and other large‐scale genomics studies.^18^, ^19^, ^20^, ^21^, ^22^ The potential of starvation‐induced genes (SIGs), such as CALR, HSPA5 and TRIB3, as molecular prognostic markers has been proposed using the TCGA database of HNSCC patients.^23^ Gene expression in HNSCC due to *P. gingivalis* infection and how changes in gene expression affect the prognosis of HNSCC patients are not clarified. Further efforts are needed to optimize the use of information in the TCGA‐HNSCC database.

The effects of *P. gingivalis* on HNSCC patients were examined herein using omics analyses. The biological activity of HNSCC cells infected with *P. gingivalis* was assessed and their RNA was sequenced. Genes showing large fluctuations in their expression in vitro were extracted and subjected to an enrichment analysis. In addition, the relationship between genes with an expression status affected by *P. gingivalis* infection and the prognosis of TCGA‐HNSCC patients was investigated. Thereby, we attempted to clarify promising genes as predictor of HNSCC associated with *P. gingivalis* infection.

## 2 MATERIALS AND METHODS

### 2.1 Bacterial and cell cultures

The bacterial strain used was *P. gingivalis* ATCC 33277. Bacteria were grown in trypticase soy broth supplemented with yeast extract (1 mg/mL), menadione (1 μg/mL) and hemin (5 μg/mL), as previously described.^14^ The human HNSCC cell line HSC‐3 was obtained from the RIKEN BRC CELL BANK (Tsukuba, Japan) and SAS cells from the Japanese Collection of Research Bioresources (Tokyo, Japan). Cultures were performed in RPMI 1640 medium (Sigma‐Aldrich, St. Louis, MO) supplemented with 10% fetal bovine serum (FBS) at 37°C in a humidified atmosphere with 5% CO_2_.

### 2.2 Bacterial adhesion assay

Bacterial adhesion assays were performed as previously described.^24^ SAS or HSC‐3 cells were infected with bacteria at a multiplicity of infection (MOI) of 1 for 90 min and then washed with phosphate‐buffered saline (PBS). To evaluate bacterial adhesion levels, cells were lysed with sterile distilled water for 15 min and dilutions of the lysate were plated and cultured anaerobically for CFU on blood agar supplemented with hemin and menadione.

### 2.3 Cell invasion assay

Cell invasion was assessed by measuring of the rate of migration of SAS and HSC‐3 cells with the BD Matrigel™ Invasion Chamber (BD Biosciences, Bedford, MA). Cells (1.0 × 10^5^) with or without *P. gingivalis* were seeded into the upper chambers in serum‐free medium, while the lower wells were filled with RPMI1640 containing 10% FBS. After the incubation, non‐invading cells were removed from the upper wells, and cells that had transferred to the inverse surface of the membrane were stained with 0.1% crystal violet. All experiments were completed in triplicate and six fields/well were counted. The invasion rate was expressed as the percentage of cells that passed through the matrigel membrane relative to migration through the control membrane (100%).

### 2.4 Immunoblot analysis

SAS and HSC‐3 cells were solubilized in cell lysis/extraction reagent (Sigma‐Aldrich, St Louis, MO) containing a protease and phosphatase inhibitor cocktail (Thermo Scientific, Rockford, IL). Immunoblotting was performed as previously described.^14^ Blots were probed with anti‐CCL20 (Thermo Scientific, Rockford, IL) and anti‐plasminogen activator urokinase (PLAU) (GeneTex International Corporation, Irvine, CA), and were reacted at 4°C overnight. The secondary antibody was alkaline phosphatase coupled with anti‐rabbit IgG (Cell Signalling Technology, Danvers, MA). Reactions were developed with the 5‐bromo‐4‐chloro‐3‐indolyl phosphate/nitroblue tetrazolium reaction. Blots were stripped and probed with an anti‐β‐actin antibody (Cell Signaling Technology) as a loading control.

### 2.5 Enzyme‐linked immunosorbent assay (ELISA) analysis

Sandwich ELISA kits were used to assess the concentrations of IL‐17 (R&D System Inc., Minneapolis, MN) and TNF‐α (R&D System Inc, Minneapolis, MN) according to the manufacturers' protocols. The SH‐1000 Lab microplate reader (Corona Electric, Ibaraki, Japan) was used to measure absorbance at 450 nm, with corrections to 550 nm.

### 2.6 RNA extraction

TRIsure (BIOLINE, Luckenwalde, Germany) was used according to the manufacturer's instructions to isolate total RNA from infected or control SAS cells. Two control samples and one 2‐h sample were prepared.

### 2.7 RNA sequencing and FASTQ file processing

The TruSeq stranded mRNA sample prep kit (Illumina, San Diego, CA) was used according to the manufacturer's protocol for library preparation. The Illumina HiSeq 2500 platform was used in the 75‐base single‐end mode for whole transcriptome sequencing. Base calling was performed using Illumina Casava ver.1.8.2 software. TopHat ver. 2.0.13 in combination with Bowtie2 ver. 2.2.3 and SAMtools ver. 0.1.19 mapped sequenced reads to human reference genome sequences (hg19). Cufflinks ver. 2.2.1 was used to calculate counts per gene. The median of the geometric means of fragment counts in all libraries was used to scale FPKMs and fragment counts.

### 2.8 Analysis of normalized count data

Normalized counts were imported into Subio Platform v1.24.5853 (Subio Inc., Aichi, Japan),^25^ which was used for all analyses. We set the lower limit by replacing positive numbers less than 10 with 10 and 0 counts with 8. The log_2_ ratio was then calculated against the geometric mean of two control samples. Genes with counts <15 or log_2_ ratios between −0.5 and 0.5 in all samples were filtered out, which left 6363 genes. Candidate DEGs were extracted using the twofold criterion.

### 2.9 Analysis of TCGA‐HNSC RNA‐Seq data

The RNA‐Seq count data of TCGA‐HNSC obtained from the GDC Data Portal^26^ were analysed with the Subio platform. The workflow of TCGA RNA‐Seq only differed from that applied to our RNA‐seq data by the thresholds used. The lower limits for positive and 0 counts were 50 and 32, respectively, while the filters on counts and log_2_ ratios were 50 and between −1 and 1, respectively. Log ratios were taken against the average of solid normal tissue samples. To compare survival times using the Kaplan–Meier method for each of the 17 selected genes, primary tumour samples were divided into those with count values higher or lower than the median.

### 2.10 Pathway and protein–protein interaction analyses

The Database for Annotation, Visualization, and Integrated Discovery server was employed for the molecular pathways of the 17 selected genes to investigate their gene ontology (GO) terms and Kyoto Encyclopedia of Genes and Genomes (KEGG) pathways. A GO enrichment analysis was performed over the following primary levels: cellular components (CC), biological processes (BP), and molecular functions (MF). A protein–protein interaction (PPI) network was established using these genes according to the STRING online database (https://string‐db.org/). We visualized the most significant modules in PPI networks.

### 2.11 Statistical analysis

Student's *t*‐test was performed using Microsoft Excel (Microsoft, Redmond, WA, USA). Results are shown as the mean ± SD. The significance of differences was set at *p* < 0.05.

The effects of PLAU gene expression (low vs. high) on all‐cause mortality was examined by a Cox proportional hazards model analysis, with PLAU gene expression as the independent variable. To correct for known risk factors, including age, sex, the HPV status, alcohol consumption, smoking (cigarettes per day) and the M, N and T stages, multivariate models were constructed with these factors also as independent variables. The proportional hazard nature of PLAU gene expression was confirmed by double log plots.

Furthermore, a Kaplan–Meier curve and the Log‐rank test using gene expression as a factor, stratified by known risk factors, were used to confirm interactions with known risk factors. Regarding interaction factors that showed significance, a Cox proportional hazards model including the interaction term was constructed to verify the effects of the interaction after corrections for confounding factors.

All statistical analyses were performed with SPSS version 24.0 for Windows (IBM Japan, Tokyo, Japan). All *p*‐values were two‐tailed; *p* < 0.05 was considered to be significant.

## 3 RESULTS

### 3.1 Effects of *P. gingivalis* on the biological activity of HNSCC cells

**FIGURE 1:**
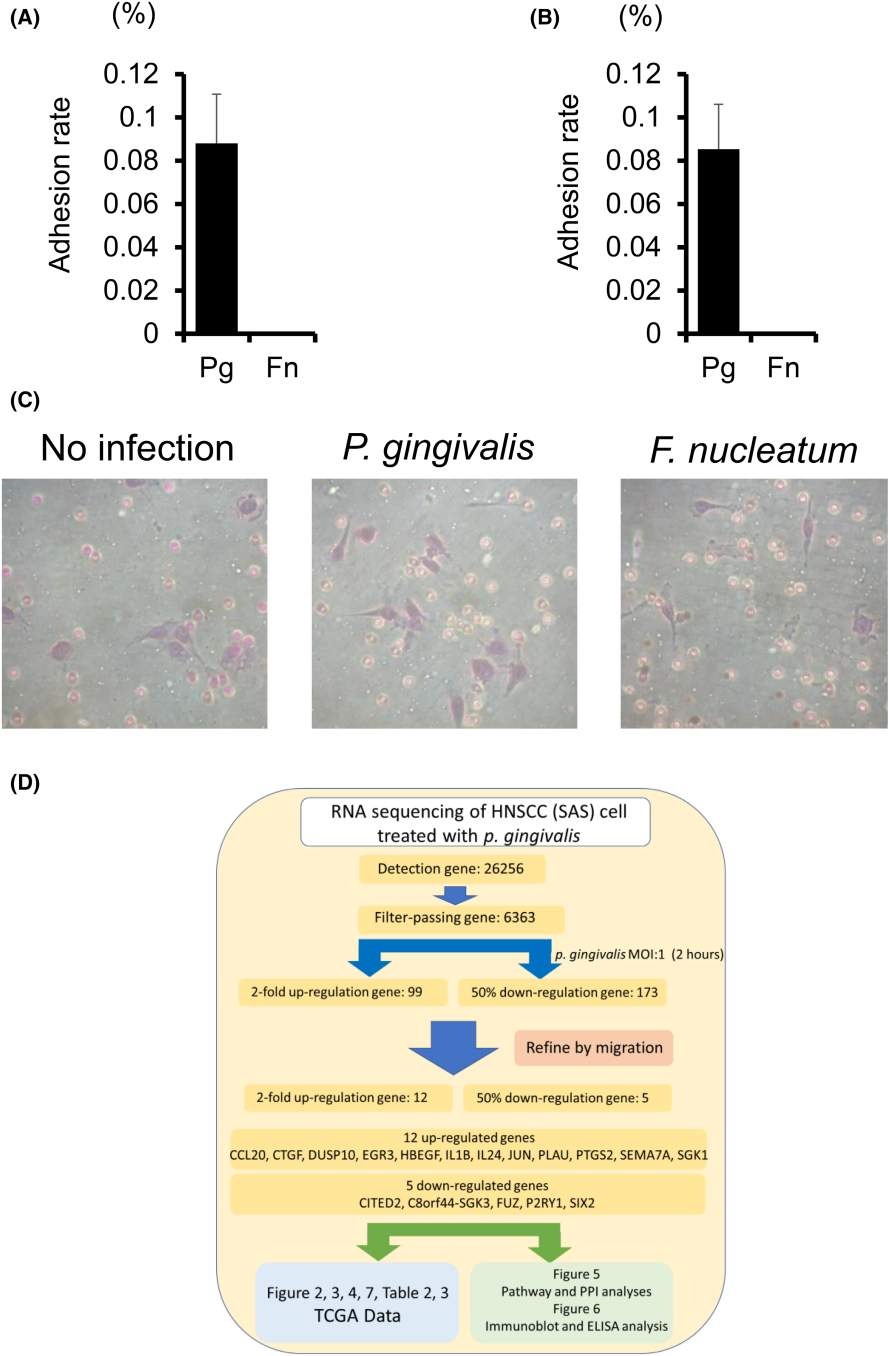
Effects of *P. gingivalis* on the biological activity of HNSCC cells. SAS cells (A) and HSC‐3 cells (B) were cultured with *P. gingivalis* or *F. nucleatum* at a MOI of 1 for 90 min. MOI 1 of *P. gingivalis* showed that adhesion to cancer cells was possible. Data are shown as the mean ± SD of 3 independent experiments and analysed by the *t*‐test. (C) SAS cell and HSC‐3 cell invasion through matrigel‐coated transwell membranes was assessed 24 h after *P. gingivalis* infection. (D) The schedule of the RNA sequencing of SAS cells after *P. gingivalis* infection.

We investigated the mechanisms underlying the responses of HNSCC cells infected with *P. gingivalis* and *F. nucleatum*. SAS cells and HSC‐3 cells were cultured with *P. gingivalis* or *F. nucleatum* at a MOI of 1 for 90 min. *P. gingivalis* was shown to adhere to SAS cells and HSC‐3 cells at significantly greater levels than *F. nucleatum* (Figure 1A,B). We previously reported on the use of matrigel‐coated transwell membranes to determine whether the adhesion efficiency of oral bacteria was related to the invasive capability of HNSCC cells.^14^ We found that cellular invasion was greater by SAS cells infected with *P. gingivalis* than by those infected with *F. nucleatum* (Figure 1C). These results are consistent with our previous finding of OSCC invasion mediated by *P. gingivalis*.^14^

### 3.2 RNA sequencing of SAS cells infected with *P. gingivalis* and changes in the expression of genes related to migration

Based on the results obtained on the biological activity of HNSCC cells infected with *P. gingivalis*, *P. gingivalis*‐infected SAS cells were subjected to RNA sequencing with or without *P. gingivalis* at a MOI of 1 for 2 h and genes showing large fluctuations in their expression were extracted. Since *P. gingivalis* is known to affect both invasion and migration,^27^ we focused on migration, which has the most extractable genes. Figure 1D shows the schedule of these experiments. After the infection of cells with *P. gingivalis* for 2 h, a more than two‐fold increase in expression was observed for 99 genes. Among them, 12 migration‐related genes, including CCL20, CTGF, DUSP10, EGR3, HBEGF, IL1B, IL24, JUN, PLAU, PTGS2, SEMA7A and SGK1, were identified (Table 1). In contrast, a 50% reduction in expression was noted in 173 genes in response to *P. gingivalis* infection. Moreover, five genes, including CITED2, C8orf44‐SGK3, FUZ, P2RY1, and SIX2, were associated with carcinoma migration (Table 1). Many of these genes were no longer altered after 24 h and the changes were transient (Table 1).

**TABLE 1:** Genes selected by the keyword migration and highly up‐ or down‐regulated after *P. gingivalis* infection.

ID	Description	NCBI Gene ID	Fold Change after 2 h	Fold Change after 24 h
SGK1	Serum/glucocorticoid regulated kinase 1	6446	2.0548851	0.84200996
CTGF	Connective tissue growth factor	1490	2.103659	1.3085716
PLAU	Plasminogen activator, urokinase	5328	2.1137452	0.972356
IL24	Interleukin 24	11009	2.1794877	0.8333334
PTGS2	Prostaglandin‐endoperoxide synthase 2	5743	2.4285715	0.4642857
CCL20	C‐C motif chemokine ligand 20	6364	2.56447	0.5604719
JUN	Jun proto‐oncogene, AP‐1 transcription factor subunit	3725	2.7633407	1.1304604
IL1B	Interleukin 1 beta	3553	2.877408	1.4091259
SEMA7A	Semaphorin 7A (John Milton Hagen blood group)	8482	3.243119	1.3338866
HBEGF	Heparin binding EGF like growth factor	1839	3.4484425	0.7531739
DUSP10	Dual specificity phosphatase 10	11221	3.7250006	1.7719301
EGR3	Early growth response 3	1960	4.2445884	0.7156118
P2RY1	Purinergic receptor P2Y1	5028	0.48148143	0.59090906
SIX2	SIX homeobox 2	10736	0.4761905	1.3000001
CITED2	Cbp/p300 interacting transactivator with Glu/Asp rich carboxy‐terminal domain 2	10370	0.42384875	1.1889815
FUZ	Fuzzy planar cell polarity protein	80199	0.33333337	0.7727273
C8orf44‐SGK3	C8orf44‐SGK3 read‐through	100533105	0.29074886	0.6567796

### 3.3 Expression of 17 genes extracted from migration keywords in TCGA‐HNSCC patients

**FIGURE 2:**
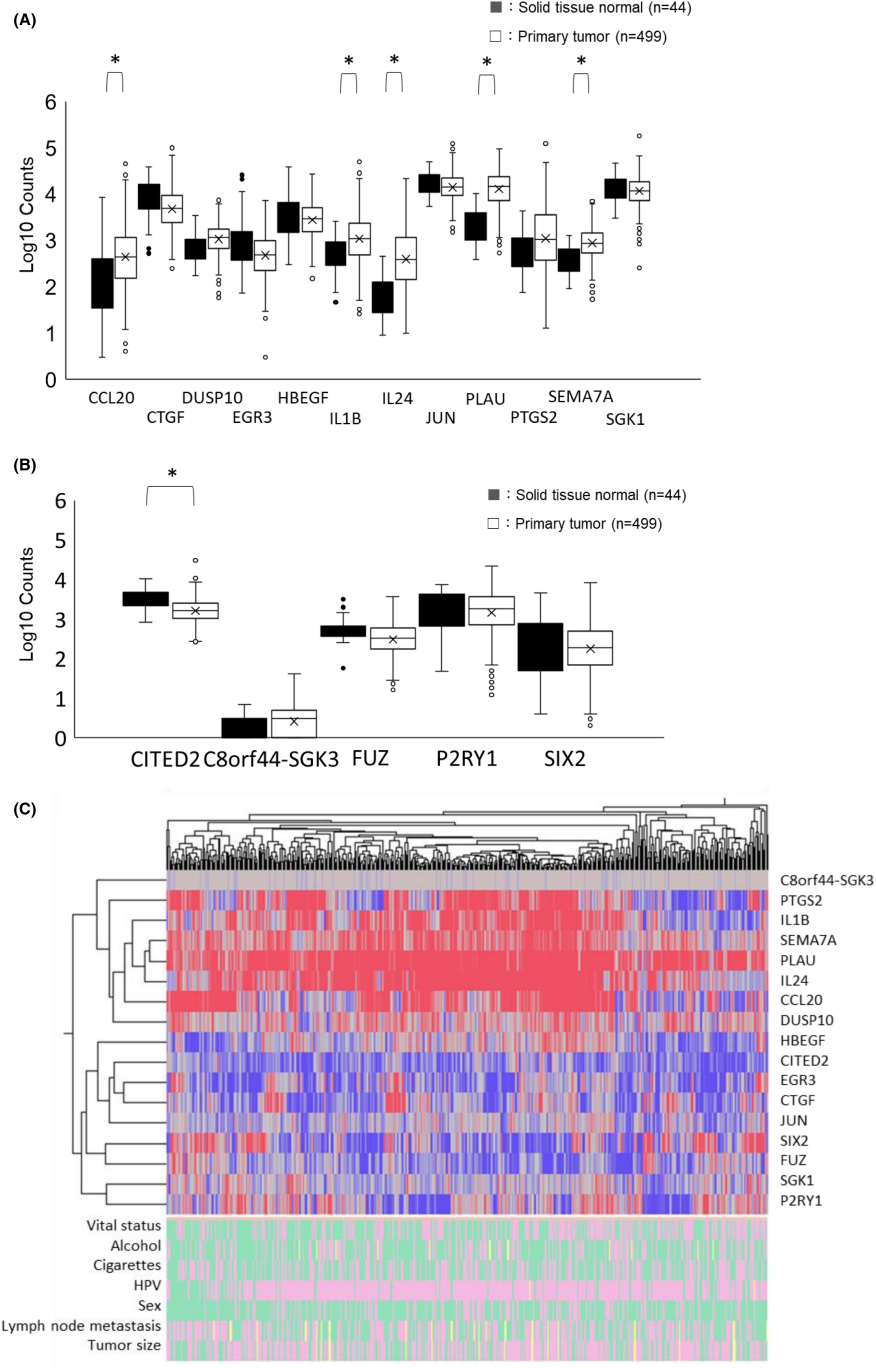
RNA sequencing of SAS cells after *P. gingivalis* infection and changes in the expression of genes related to migration. The expression of 17 genes showed significant changes after *P. gingivalis* infection and they were extracted by migration keywords. Their relative expression levels in TCGA‐HNSCC patients were assessed in primary tumours and solid normal tissues. (A) Box plots of the expression of 12 genes (CCL20, CTGF, DUSP10, EGR3, HBEGF, IL1B, IL24, JUN, PLAU, PTGS2, SEMA7A, and SGK1) showing more than two‐fold increases in expression. (B) Box plots of the expression of 5 genes (CITED2, C8orf44‐SGK3, FUZ, P2RY1, and SIX2) showing a 50% reduction in expression. (C) Heat map of the 17 gene expression profiles. Colours from blue to red indicate low to high expression levels. The detailed clinical data breakdown of the patients is as follows: alive ( 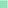 ), dead ( 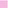 ), alcohol yes ( 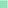 ), alcohol no ( 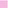 ), alcohol unknown ( 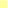 ), cigarettes yes ( 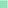 ), cigarettes no ( 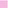 ), HPV positive ( 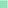 ), HPV negative ( 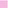 ), HPV unknown ( 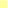 ), Male ( 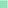 ), Female ( 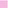 ), lymph node metastasis yes ( 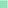 ), lymph node metastasis no ( 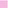 ), lymph node metastasis unknown ( 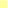 ), tumour size T1‐2 ( 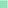 ), tumour size ≥T3 ( 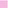 ), tumour size unknown ( 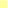 ).

Gene expression was examined in the tumour tissues of TCGA‐HNSCC patients. We focused on 17 genes associated with migration in cells infected with *P. gingivalis*. In clinical samples analysed by TCGA, the expression of 5 genes, CCL20, IL1B, IL24, PLAU and SEMA7A, was significantly higher in the primary tumour than in solid normal tissue (Figure 2A), whereas that of CITED2 was lower (Figure 2B). A heat map was also created to represent the degree to which the expression of the 17 genes was up‐ or down‐regulated (Figure 2C). The detailed clinical data breakdown of the TCGA‐HNSCC 499 patients used in this study was as follows: alive (*n* = 282; 56.5%), dead (*n* = 217; 43.5%), alcohol yes (*n* = 332; 66.5%), alcohol no (*n* = 156; 31.3%), alcohol unknown (*n* = 11; 2.2%), cigarettes yes (*n* = 286; 57.3%), cigarettes no (*n* = 213; 42.7%), HPV positive (*n* = 89; 17.8%), HPV negative (*n* = 408; 81.8%), HPV unknown (*n* = 2; 0.4%), male (*n* = 367; 73.5%), female (*n* = 132; 26.5%), lymph node metastasis yes (*n* = 239; 47.9%), lymph node metastasis no (*n* = 238; 47.7%), lymph node metastasis unknown (*n* = 22; 4.4%), tumour size T1‐2 (*n* = 176; 35.3%), tumour size ≥T3 (*n* = 308; 61.7%), tumour size unknown (*n* = 15; 3.0%). The heat map also included detailed clinical data on vital status, drinking, smoking, the HPV status, sex, the presence of lymph node metastasis and tumour size (Figure 2C).

### 3.4 Prognostic significance of 17 genes extracted from migration keywords in TCGA‐HNSCC patients

**FIGURE 3:**
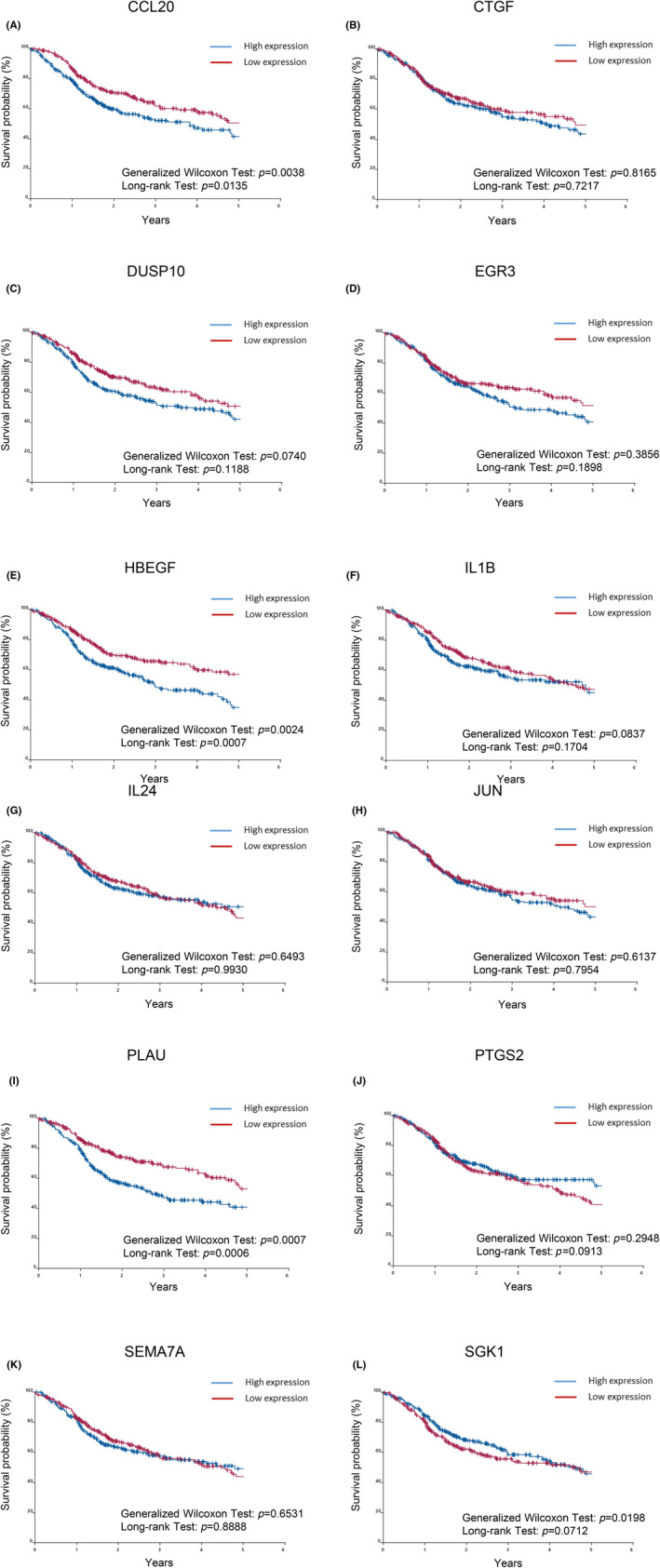
Prognostic significance of 12 up‐regulated genes extracted from migration keywords in TCGA‐HNSCC patients. The overall survival of TCGA‐HNSCC patients, classified by 12 up‐regulated genes extracted from migration keywords, was assessed by the Kaplan–Meier method. Differences in survival times measured by the Kaplan–Meier method were examined using the generalized Wilcoxon test and Log rank test. A: CCL20, B: CTGF, C: DUSP10, D: EGR3, E: HBEGF, F: IL1B, G: IL24, H: JUN, I: PLAU, J: PTGS2, K: SEMA7A, L: SGK1.

**FIGURE 4:**
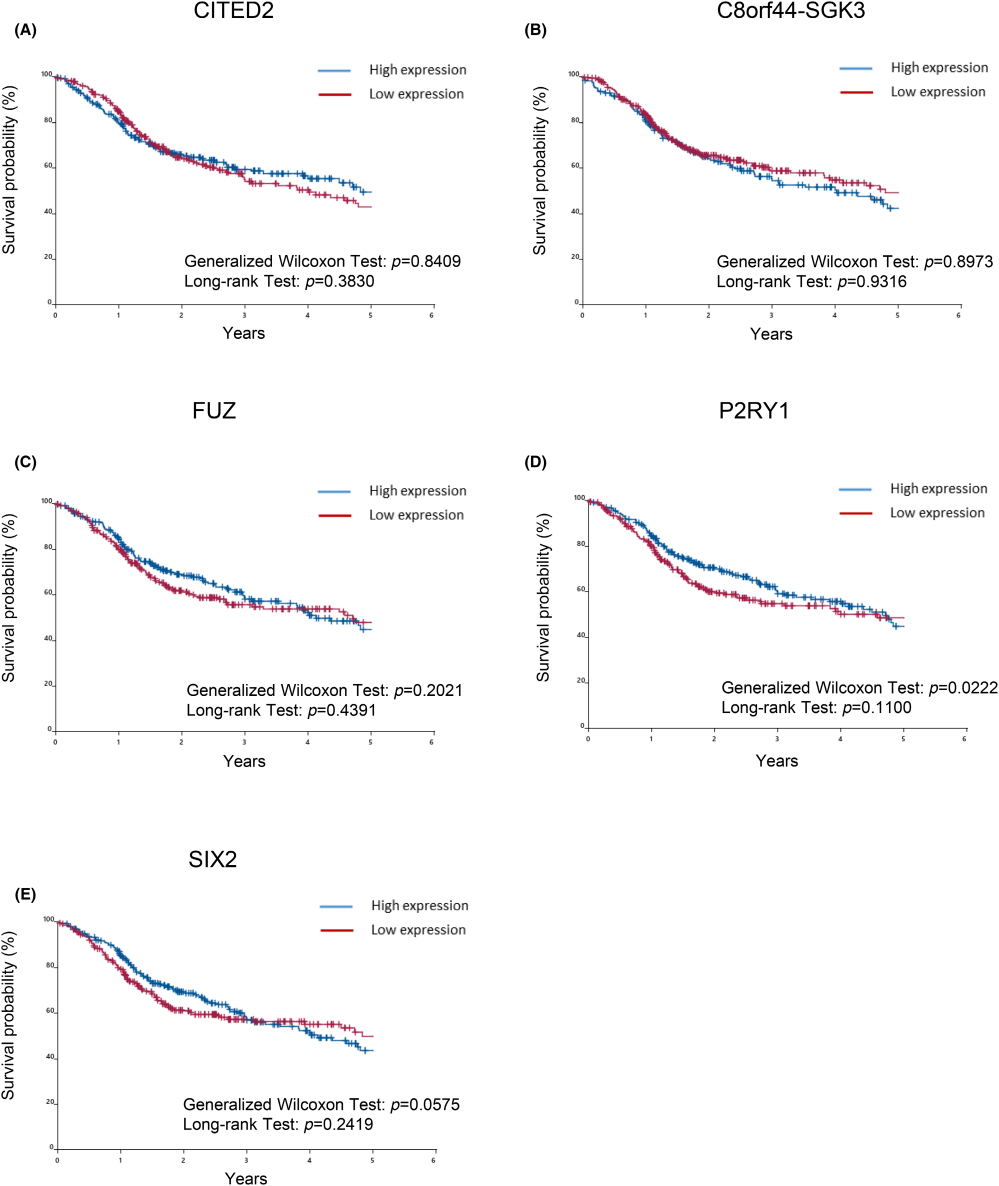
Prognostic significance of 5 down‐regulated genes extracted from migration keywords in TCGA‐HNSCC patients. The overall survival of TCGA‐HNSCC patients, classified by 5 down‐regulated genes extracted from migration keywords, was assessed by the Kaplan–Meier method. Differences in survival times measured by the Kaplan–Meier method were examined using the generalized Wilcoxon test and Log rank test. A: CITED2, B: C8orf44‐SGK3, C: FUZ, D: P2RY1, E: SIX2.

We investigated whether the 17 genes associated with migration listed in Table 1 affected the prognosis of patients. Patients were divided into two groups based on the high or low expression of these genes. Expression levels higher than the median were classified as the high expression group and the remainder as the low expression group.^23^, ^28^, ^29^ The generalized Wilcoxon test and Log rank test were performed to assess the differences in survival times identified by the Kaplan–Meier method (Figures 3 and 4). Among the 12 up‐regulated genes, the high expression of CCL20 (Figure 3A), HBEGF (Figure 3E), and PLAU (Figure 3I) correlated with shorter survival times, whereas the high expression of CTGF (Figure 3B), DUSP10 (Figure 3C), EGR3 (Figure 3D), IL1B (Figure 3F), IL24 (Figure 3G), JUN (Figure 3H), PTGS2 (Figure 3J), SEMA7A (Figure 3K) and SGK1 (Figure 3L) did not. Down‐regulated genes were also examined using the Kaplan–Meier method, which revealed no relationship between gene expression and survival in TCGA‐HNSCC patients (Figure 4A–E).

### 3.5 Function and PPI analyses of 17 genes extracted from migration keywords

**FIGURE 5:**
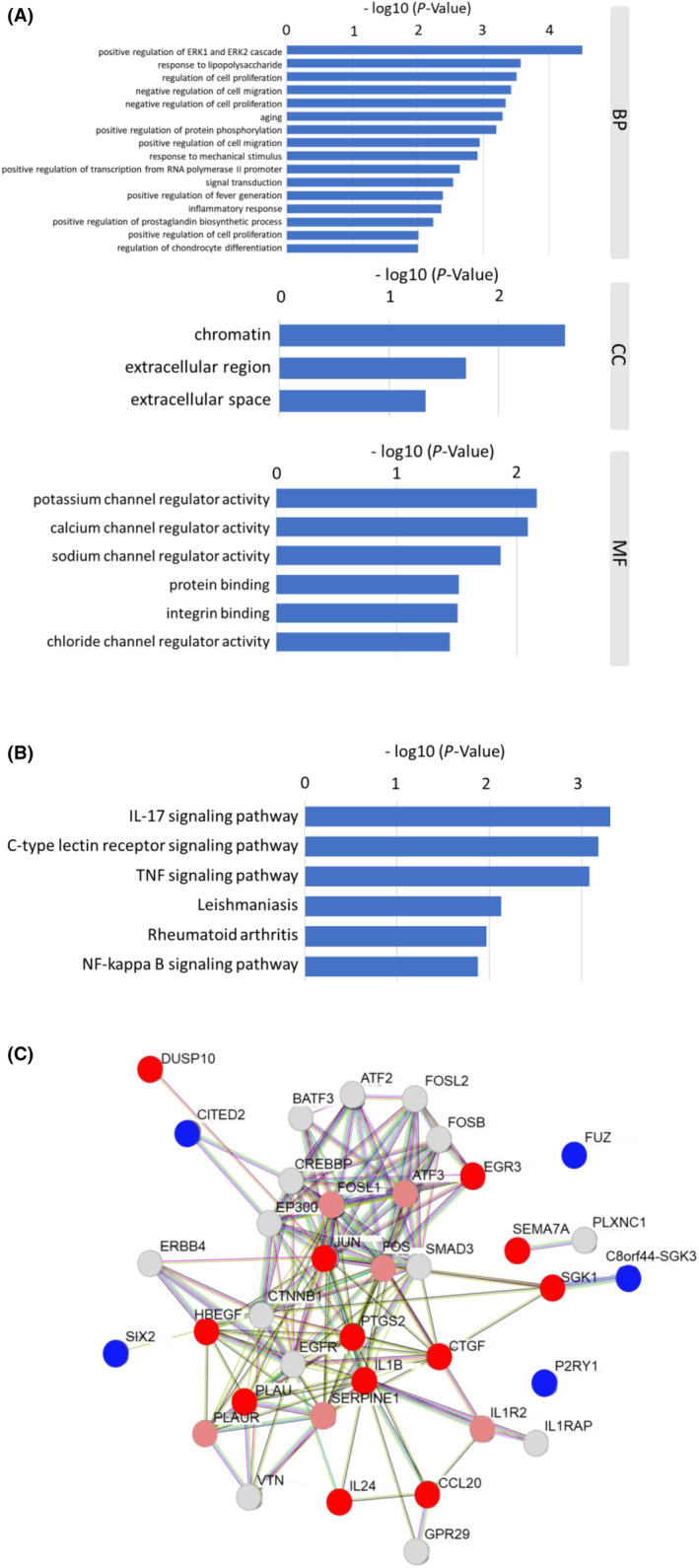
Function and PPI analyses of 17 genes extracted from migration keywords. (A) A list of GO terms identified by the GO enrichment analysis of 17 selected genes. BP, biological process; CC, cellular composition; MF, molecular function. (B) List of molecular pathways identified by the KEGG pathway enrichment analysis of 17 selected genes. (C) Proteins encoded by 17 genes extracted from migration keywords were subjected to a PPI network analysis. Up‐regulated genes are shown in red. Down‐regulation is shown in blue, and grey indicates genes with expression levels that remained unchanged after *P. gingivalis* infection.

The biological properties and potential signalling pathways of the 17 selected genes were investigated using GO and KEGG enrichment pathway analyses. The GO enrichment analysis identified the following enriched terms: positive regulation of the ERK1/2 cascade, response to lipopolysaccharide, regulation of cell proliferation, negative regulation of cell migration, negative regulation of cell proliferation, aging, positive regulation of protein phosphorylation, positive regulation of cell migration, response to mechanical stimulus, positive regulation of transcription from RNA polymerase II promoter, signal transduction, positive regulation of fever generation, inflammatory response, positive regulation of prostaglandin biosynthetic process, positive regulation of cell proliferation and regulation of chondrocyte differentiation (Figure 5A). The KEGG analysis revealed the significant enrichment of prognostic genes in the following pathways: IL‐17 signalling pathway, C‐type lectin receptor signalling pathway, TNF signalling pathway, Leishmaniasis, Rheumatoid arthritis and NF‐kappa B signalling pathway (Figure 5B). In the PPI network analysis, ATF2, ATF3, BATF3, CCL20, CREBBP, CTGF, CTNNB1, EP300, EGFR, EGR3, ERBB4, FOS, FOSB, FOSL1, FOSL2, GPR29, HBEGF, IL1R2, IL24, JUN, PLAU, PLAUR, PTGS2, SERPINE1, SMAD3 and VTN formed a close interaction network (Figure 5C).

### 3.6 Expression levels of CCL20, PLAU, IL17 and TNF‐α

**FIGURE 6:**
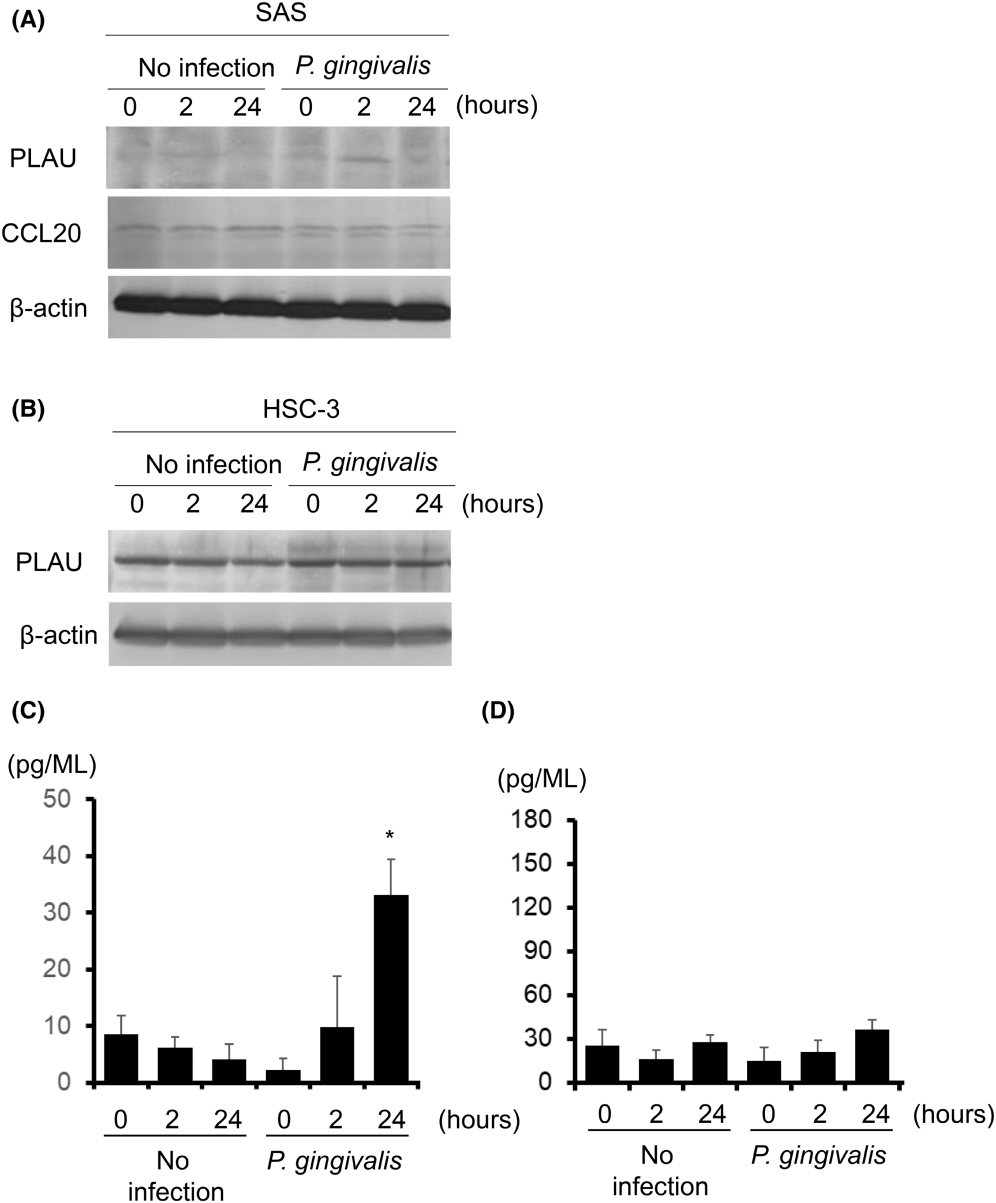
Expression levels of CCL20, PLAU, IL17, and TNF‐α. Cells were incubated with *P. gingivalis* at a MOI of 1 for the indicated times, and lysates were subjected to immunoblotting. (A) PLAU and CCL 20 (SAS cells), (B) PLAU (HSC‐3 cells). β‐Actin was included as a loading control for whole cell lysates. ELISA showed IL‐17 production (C) and TNF‐α production (D) using SAS cells.

The expression of CCL20 and PLAU significantly differed between primary tumours and solid normal tissues and in survival curves by the Kaplan–Meier method (Figure 3A,I). Therefore, we examined the expression of CCL20 and PLAU, and each was confirmed to be expressed in SAS cells. The most efficient expression level of PLAU in SAS cells was observed at 2 h following *P. gingivalis* infection, but its expression was decreased at 24 h (Figure 6A). In HSC‐3 cells, basal levels of PLAU were higher than in SAS cells and were not specifically altered by *P. gingivalis* infection (Figure 6B). Similar expression levels were maintained in HSC‐4 cells derived from HSC‐3 cells (data not shown). Expression levels of CCL20 in SAS cells did not change with time (Figure 6A). We then examined the TNF‐α and IL‐17 signalling pathways extracted by the KEGG analysis (Figure 5B). ELISA showed that IL‐17 production increased in SAS cells infected with *P. gingivalis* (Figure 6C), while TNF‐α production remained unchanged (Figure 6D).

### 3.7 Relationship of PLAU and classical prognostic factors with survival in TCGA‐HNSCC patients

**FIGURE 7:**
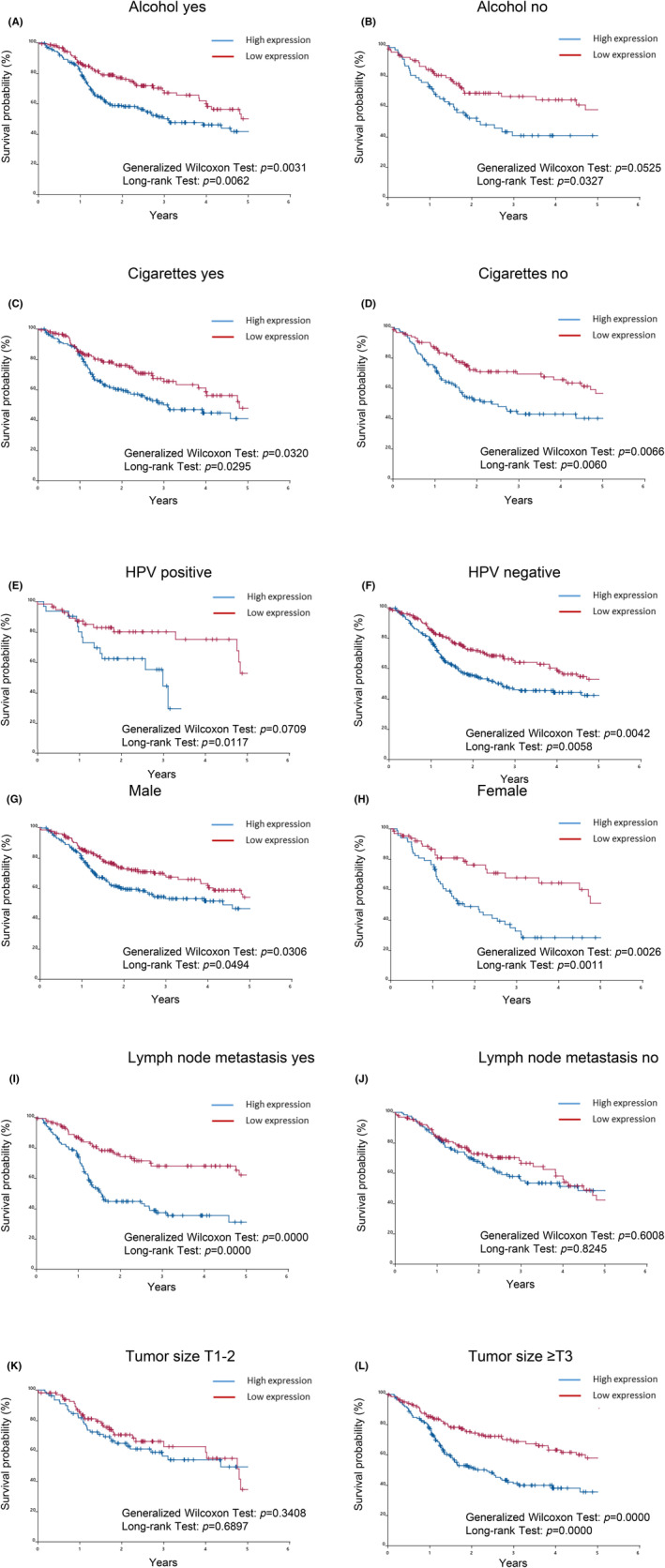
The relationship of PLAU and classical prognostic factors with survival in TCGA‐HNSCC patients. The survival curve was recalculated based on the expression of PLAU, in consideration of classical prognostic factors, such as drinking, smoking, the HPV status, sex, the presence of lymph node metastasis, and tumour sizes. (A) Groups with a history of drinking. (B) Groups without a history of drinking. (C) Groups with a history of smoking. (D) Groups without a history of smoking. (E) Groups with a history of HPV infection. (F) Groups without a history of HPV infection. (G) Male group. (H) Female group. (I) Groups with lymph node metastasis. (J) Groups without lymph node metastasis. (K) Groups with tumour sizes T1‐T2. (L) Groups with tumour sizes ≥T3.

Since high PLAU expression correlated with shorter survival (Figure 3I) and increases in PLAU due to the *P. gingivalis* infection were also observed at the protein level (Figure 6A), the effects of drinking (Figure 7A,B), smoking (Figure 7C,D), the HPV status (Figure 7E,F), sex (Figure 7G,H), the presence of lymph node metastasis (Figure 7I,J), and tumour size (Figure 7K,L) on PLAU patient survival were examined. Survival curves were not affected by PLAU expression in the absence of lymph node metastasis (Figure 7J), but were markedly changed by its expression in cases with lymph node metastasis (Figure 7I). Furthermore, survival curves were not affected by PLAU expression in T1‐T2 tumours (Figure 7K), but were significantly changed by its expression in tumours ≥T3 (Figure 7L).

### 3.8 Cox regression analysis of the relationship of PLAU and classical prognostic factors with survival in TCGA‐HNSCC patients

Table 2 shows the effects of PLAU gene expression on all‐cause mortality. The univariate analysis showed a higher risk of all‐cause mortality in the high PLAU expression group than in the low PLAU expression group (hazard ratio [HR] = 1.722 [95%CI: 1.294, 2.293], *p* < 0.001). Among known risk factors, age, sex, the M stage and N stage correlated with all‐cause mortality. The multivariate analysis to correct for confounding factors showed that high PLAU expression was a significant risk factor for all‐cause mortality even after adjustments for known risk factors (HR = 1.804 [95%CI: 1.331, 2.446], *p* < 0.001).

**TABLE 2:** Univariate and multivariate analyses of the PLAU gene using TCGA‐HNSC patient data.

	Univariate			Multivariate		
	HR	95% CI	*p*‐value	HR	95% CI	*p*‐value
Without the interaction mode						
PLAU_High (vs. Low)	1.722	1.294–2.293	**<0.001**	1.804	1.331–2.446	**<0.001**
Age (per 1 year)	1.016	1.003–1.029	**0.015**	1.023	1.009–1.038	**0.001**
Sex_male (vs. female)	0.721	0.535–0.973	**0.032**	0.873	0.620–1.228	0.434
HPV status_Positive (vs. Negative)	0.777	0.524–1.153	0.210	0.813	0.528–1.252	0.348
Drinking_history_Yes (vs. No)	0.947	0.701–1.279	0.723	1.021	0.732–1.425	0.902
Cigarettes per day_>0 (vs. 0)	0.960	0.724–1.272	0.774	0.944	0.699–1.276	0.709
M stage_m1 (vs. m0)	4.748	1.749–12.889	**0.002**	5.096	1.774–14.638	**0.002**
N stage (per 1 category)	1.078	1.002, 1.160	**0.045**	1.112	1.026–1.206	**0.010**
T stage (per 1 category)	0.999	0.902, 1.106	0.986	0.991	0.888–1.105	0.866

*Note*: Bold type indicates p <0.05.

A stratified Kaplan–Meier analysis suggested interactions between lymph node metastasis, tumour size and PLAU expression (Figure 7I–L). The results of the multivariate model testing these interaction terms under corrections for confounding factors are shown in Table 3.

**TABLE 3:** Multivariate analyses with the interaction mode of the PLAU gene using TCGA‐HNSC patient data.

	Multivariate		
	HR	95% CI	*p*‐value
With the interaction mode [PLAU × Lymph node metastasis]			
Age (per 1 year)	1.019	1.005–1.033	**0.006**
Sex_male (vs. female)	0.832	0.607–1.141	0.254
M stage_m1 (vs. m0)	3.231	1.169–8.932	**0.024**
Lymph node metastasis. yes (vs. no)	0.739	0.459–1.190	0.213
PLAU_High (vs. Low) [in lymph node metastasis_yes]	2.908	1.864–4.536	**<0.001**
PLAU_High (vs. Low) [in lymph node metastasis_no]	1.189	0.789–1.792	0.407
PLAU × lymph node metastasis	2.445	1.336–4.474	**0.004**
With the interaction mode [PLAU × tumour size]			
Age (per 1 year)	1.018	1.005–1.032	**0.007**
Sex_male (vs. female)	0.766	0.560–1.049	0.097
M stage_m1 (vs. m0)	4.587	1.666–12.626	**0.003**
Tumour size_ ≥ T3 (vs. T1‐2)	0.779	0.490–1.240	0.293
PLAU_High (vs. Low) [in tumour size_ ≥ T3]	2.298	1.595–3.312	**<0.001**
PLAU_High (vs. Low) [in tumour size_ ≥ T3]	1.043	0.629–1.728	0.870
PLAU × tumour size_ ≥ T3	2.204	1.182– 4.109	**0.013**

*Note*: Confounding factors to be corrected for in this analysis are background factors that were significant in the multivariate analysis in Table 2 (age, sex and the M stage). Bold type indicates *p* < 0.05.

The results of the interaction analysis with lymph node metastasis showed that PLAU expression was a significant factor affecting all‐cause mortality only in the population with lymph node metastasis (HR = 2.908 [95%CI: 1.864, 4.536], *p* < 0.001), and the interaction term was also significant (*p* = 0.004 [PLAU × Lymph node metastasis]).

The results of the interaction analysis with tumour size showed that PLAU expression was a significant factor affecting all‐cause mortality only in the population with a large tumour size (≥T3) (HR = 2.298 [95%CI: 1.595, 3.312], *p* < 0.001), and the interaction term was also significant (*p* = 0.013 [PLAU × Tumour size]).

## 4 DISCUSSION

Periodontitis, a bacterial gingival disease, is associated with a two‐ to fivefold higher risk of developing cancer, particularly *P. gingivalis* and *F. nucleatum*, which were found to be metastatic by several different mechanisms.^30^, ^31^ In addition, periodontitis was also shown to be predictive of a 5.23‐fold increase in the risk of tongue cancer.^14^, ^32^ *P. gingivalis*, a major periodontal pathogen, has been identified as a specific and potentially independent microbial factor increasing the risk of orodigestive cancer mortality.^16^, ^33^ In the present study, *P. gingivalis* adhered to HNSCC cells at significantly higher levels than *F. nucleatum* at the same MOI. We previously reported that *P. gingivalis* promoted the migration and invasion of HNSCC cell lines through the induction and activation of proMMP9, whereas the same periodontal pathogen, *F. nucleatum*, failed to exhibit this activity. These findings suggested that *P. gingivalis* has the specific property of adhering to HNSCC cells and changing their behaviour.

A GO analysis, KEGG pathway enrichment analysis, and construction of a PPI network have been used to identify key genes and pathways involved in carcinogenesis and cancer progression.^23^, ^34^ Geng et al. reported that L6, STAT1, LYN, BDNF, C3,CD274, PDCD1LG2 and CXCL10 were important candidates associated with OSCC using bioinformatical analyses and attempted to demonstrate that *P. gingivalis* infection promoted the initiation and progression of OSCC.^35^ In the present study, the GO analysis extracted 16 biological processes, including the ERK1/2 cascade, response to lipopolysaccharide, regulation of cell proliferation, and negative regulation of cell migration. KEGG analyses also identified 6 pathways, including IL‐17 signalling pathway, C‐type lectin receptor signalling pathway, TNF signalling pathway, Leishmaniasis, Rheumatoid arthritis, and NF‐kB signalling pathway, in *P. gingivalis*‐mediated HNSCC metastasis that may be relevant to *P. gingivalis* infection in HNSCC. We previously reported that *P. gingivalis* activated the ERK1/2‐Ets1, p38/HSP27 and PAR2/NF‐kB pathways to induce proMMP9 expression, after which the proenzyme was activated by gingipains to promote cellular invasion by HNSCC cell lines.^14^ The activation of these pathways was consistent with the results of the GO and KEGG enrichment pathway analyses. However, the involvement of IL‐17‐ and TNF‐α‐related pathways by *P. gingivalis* infection has yet to be demonstrated. The IL‐17 family of cytokines consists of six groups: IL‐17A, IL‐17B, IL‐17C, IL‐17D, IL‐17E and IL17‐F, which are involved in the development of autoimmunity, inflammation, tumours, host defences against bacterial and fungal infections, and mucosal host defence mechanisms.^36^, ^37^ In the present study, IL‐17 production was increased by *P. gingivalis* infection (Figure 5B). This result suggests that *P. gingivalis* infection activates IL‐17 pathways, leading to the development of and metastasis by HNSCC. CCL20 has been reported to indirectly promote tumour progression by recruiting Treg, Th17 and Th22 cells in order to maintain a microenvironment of development and immunosuppression.^38^, ^39^ CCL20 was also found to be associated with *P. gingivalis* infection in HNSCC.

PLAU, also known as urokinase‐type plasminogen activator, is a serine protease involved in tissue remodelling and cell migration.^40^, ^41^ PLAU is considered to play an important role in wound healing, neuronal migration, and vascular remodelling.^42^, ^43^ Furthermore, in malignant tumours, PLAU is involved in tumorigenesis, progression, cell invasion, and angiogenesis.^40^, ^42^ The higher expression of PLAU is regarded as a poor prognostic marker in aggressive breast cancer, hepatocellular carcinoma, and gastric cancer.^44^, ^45^, ^46^ In the present study, we identified genes related to *P. gingivalis*‐induced migration, including 12 up‐ or 5 down‐regulated genes, by an in vitro assay using the TCGA database. Moreover, CCL20 and PLAU were suggested by the Kaplan–Meier method. PLAU has been associated with the prognosis of patients with head and neck cancer.^47^, ^48^, ^49^, ^50^ In a HNSCC bioinformatics study, PLAU, SERPINE1, and ACTA1 were linked to the prognosis of patients. Among them, the labelling score of PLAU significantly increased from well (106.78 ± 37.323%) through to moderately (182.40 ± 6.450%) and poorly differentiated HNSCC samples (272.50 ± 36.812%).^47^ Immunohistochemical staining revealed that PLAU was expressed mainly in the cytoplasm, with increased expression in HNSCC compared to normal oral mucosa.^47^ In addition, the up‐regulated expression of PLAU was associated with HPV positivity and the neck node status.^50^ ITGA5, PLAU, PLAUR, SERPINE1 and TGFB1 have potential as prognostic biomarkers for HNSCC mediated by HPV infection.^48^ Expression of PLAU in HNSCC was also detected by immunohistochemical staining. These previous studies and the present study demonstrate the importance of PLAU in HNSCC. However, this study is probably the first to show that PLAU is one of the genes whose expression is up‐regulated in the pathway where *P. gingivalis* infection increases HNSCC cell motility and is a candidate biomarker for decreased survival in HNSCC patients. Although PLAU has been associated with a malignant status, the activation of the PLAU proteolytic cascade has been implicated in alveolar bone loss in *P. gingivalis*‐mediated periodontal disease.^51^ In the present study, we attempt to detect changes in PLAU protein expression due to *P. gingivalis* infection and found a transient increase of PLAU protein (Figure 6A). However, no significant increase was observed in HSC‐3 cells. The higher baseline levels of PLAU in HSC‐3 cells may make it difficult to detect changes in PLAU protein. Previously, we reported that proMMP‐9 was produced and activated in SAS cells infected with *P. gingivali*s, but not other oral SCC cell line.^14^ These findings indicate the heterogeneity of HNSCC cells, which may result in the different behaviour of each HNSCC cell line against specific bacteria. Nevertheless, it is clear that the findings with SAS cells provide valuable clues to begin investigating candidate of prognostic biomarkers through the TCGA HNSCC database. To correlate in vitro findings in HNSCC cell lines with those in TCGA tumour samples, it is better to infect HNSCC sample tissue with *P. gingivalis* and compare gene expression in infected and uninfected tumour tissues. Future experiments using such an experimental model will provide information on the response of HNSCC tumours to *P. gingivalis* and will help to validate in vitro studies with HNSCC cell lines.

The etiological factors correlating with HNSCC include tobacco use, alcohol consumption, and HPV infection.^22^, ^52^ In addition, lymph node metastasis and tumour size have been associated with its prognosis.^53^ We investigated the relationship between PLAU and these factors, such as drinking, smoking, the HPV status, sex, the presence of lymph node metastasis and tumour size, because the expression level of PLAU was also confirmed by an immunoblot analysis. The univariate analysis showed a higher risk of all‐cause mortality in the high PLAU expression group than in the low PLAU expression group. Among known risk factors, age, sex, the M stage and N stage correlated with all‐cause mortality. The multivariate analysis to correct for confounding factors showed that high PLAU expression was a significant risk factor for all‐cause mortality even after adjusting for known risk factors. The interaction analysis with lymph node metastasis identified PLAU expression as a significant factor affecting all‐cause mortality only in the population with lymph node metastasis. The interaction analysis with tumour size also showed that PLAU expression was a significant factor affecting all‐cause mortality only in the population with a large tumour size (≥T3), and the interaction term was also significant. These results suggest that PLAU is a prognostic factor, similar to the M stage and N stage. Survival rates for patients with no lymph node metastases or T1‐2 tumours, regardless of PLAU expression, were equivalent to those of patients with low PLAU expression and large tumours or lymph node metastases (Figure 7I–L). Therefore, if PLAU expression due to *P. gingivalis* infection is down‐regulated, the risk of death in patients with advanced cancer may be reduced to that in patients with T1‐2 tumours or without lymph node metastases. These findings indicate the possibility that PLAU serve as a candidate prognostic biomarker for HNSCC patients infected with *P. gingivalis*. Future clinical studies are expected to determine whether oral hygiene is related to the efficacy of treatment of HNSCC in the PLAU high‐expression group, and what happens to PLAU expression when oral hygiene is improved.

In conclusion, Kaplan–Meier and Cox analyses of PLAU expression with *P. gingivalis* infection showed the potential of PLAU as a molecular prognostic marker in advanced HNSCC patients with tumour sizes ≥T3 or advanced HNSCC patients with lymph node metastases. Further in vivo and in vitro studies are needed to verify the findings of this study.

## AUTHOR CONTRIBUTIONS

**Masakazu Hamada:** Conceptualization (equal); data curation (equal); formal analysis (equal); funding acquisition (equal); investigation (equal); methodology (equal); validation (equal); visualization (equal); writing – original draft (equal); writing – review and editing (equal). **Hiroaki Inaba:** Conceptualization (equal); data curation (equal); formal analysis (equal); funding acquisition (equal); investigation (equal); methodology (equal); validation (equal); visualization (equal); writing – original draft (equal); writing – review and editing (equal). **Kyoko Nishiyama:** Formal analysis (equal); funding acquisition (equal); investigation (equal); methodology (equal); visualization (equal); writing – review and editing (equal). **Sho Yoshida:** Formal analysis (equal); investigation (equal); methodology (equal); writing – review and editing (equal). **Yoshiaki Yura:** Conceptualization (equal); formal analysis (equal); methodology (equal); supervision (equal); writing – original draft (equal); writing – review and editing (equal). **Michiyo Matsumoto‐Nakano:** Conceptualization (equal); formal analysis (equal); supervision (equal); writing – review and editing (equal). **Narikazu Uzawa:** Conceptualization (equal); formal analysis (equal); supervision (equal); writing – review and editing (equal).

## CONFLICT OF INTEREST STATEMENT

The authors declare no conflicts of interest.

## Data Availability

The data are available from the corresponding author upon reasonable request.
